# Activation of the COX2-PGE_2 _axis by immune effector cells results in the self-limiting nature of type-1 immunity in cancer tissues

**DOI:** 10.1186/2051-1426-2-S3-P239

**Published:** 2014-11-06

**Authors:** Jeffrey L Wong, Natasa Obermajer, Ravikumar Muthuswamy, Robert P Edwards, Kunle Odunsi, David L Bartlett, Pawel Kalinski

**Affiliations:** 1 Dept of Surgery, University of Pittsburgh School of Medicine, Pittsburgh, PA, USA; 2 Magee-Womens Research Institute, Pittsburgh, PA, USA; 3 Dept of Gynecologic Oncology, Roswell Park Cancer Institute, Buffalo, NY, USA

## 

Type-1 immune responses, mediated by IFNγ and TNFα-producing CTLs, Th1, and NK cells, are essential for effective anti-tumor immunity. Despite recent advances in the induction and stabilization of these responses by cancer immunotherapies, the clinical success of these approaches remain limited. Here, we report that the activation of type-1 immunity within the human tumor microenvironment (TME) initiates IFNγ- and TNFα-dependent counter-regulation, driven by amplification of prostaglandin E_2 _(PGE_2_) and the key regulator of PGE_2 _synthesis, cyclooxygenase 2 (COX2). We demonstrate that type-1-activated CTLs and NK cells induce IFNγ/TNFα-mediated over-expression of indoleamine 2,3-dioxygenase (IDO), inducible nitric oxide synthase (iNOS/NOS2), IL-10, and COX2 by tumor-associated myeloid-derived suppressor cells (MDSCs). Importantly, this self-limiting suppressive feedback driven by type-1 immunity could be eliminated not only by neutralization of IFNγ and TNFα, the factors critically required for the anti-tumor activity of immune effector cells, but also by COX2 blockade, which counteracted the IFNγ/TNFα-driven enhancement of all other suppressive factors, amplifying the therapeutic potential of intratumoral type-1 immunity. Our data demonstrate an intrinsic mechanism underlying the self-limiting character of type-1 immunity within the human TME, and provide rationale for targeting the COX2-PGE_2 _axis to enhance desirable type-1 responses for cancer immunotherapy.

